# The prevalence of ESBL-producing Enterobacteriaceae in a nursing home setting compared with elderly living at home: a cross-sectional comparison

**DOI:** 10.1186/s12879-016-1430-5

**Published:** 2016-03-04

**Authors:** Andreas Blom, Jonas Ahl, Fredrik Månsson, Fredrik Resman, Johan Tham

**Affiliations:** Infectious Diseases Research Unit, Deptment of Clinical sciences, Lund University, Skånes University hospital, 205 02 Malmö, Sweden

**Keywords:** Elderly, ESBL, Nursing home, Enterobacteriaceae

## Abstract

**Background:**

The aim of the study was to investigate the prevalence of faecal carriage of extended-spectrum beta-lactamase (ESBL)-producing *Enterobacteriaceae* among residents living in nursing homes and to compare it with a corresponding group of elderly people living in their own homes.

**Methods:**

A total of 160 persons participated in the study between February and April 2014, 91 were residents in nursing homes (*n* = 10) and the remaining 69 were elderly living in their own homes. In addition to performing faecal samples, all participants answered a standardized questionnaire regarding known risk factors for ESBL-carriage.

**Results:**

There was no significant difference between the groups, as 10 of the 91 (11 %) residents from nursing homes were ESBL-carriers compared with 6 of 69 (8,7 %) elderly living in their own homes. There was no significant difference between the groups. The total prevalence was 10 %. A univariate analysis revealed that the only studied risk factor significantly associated with ESBL-carriage was recent foreign travel (*p* = 0,017). All ESBL-positive isolates were *Escherichia coli* and there was a high degree of co-resistance to other antibiotics. All isolates (*n* = 17) were susceptible to imipenem and amikacin.

**Conclusion:**

Residents of nursing homes as well as elderly living in their own homes have high rates of faecal carriage of ESBL-producing bacteria. These findings may affect the choice of empirical antibiotic treatment of severe infections in older adults.

## Background

The rapid increase of ESBL-producing bacteria is a growing problem, and has been described as a pandemic [1]. Reported risk factors for carriage and infection include antibiotic treatment, age ≥ 65 years, recent hospitalisation, prolonged hospital stay, recent surgery, recurrent UTIs, travel to foreign countries, severe illness, immobilization and residence at nursing home [2–8]. However, ESBL-carriage occurs in patients with no apparent risk factors as well [2, 9]. Age is a risk factor, but a Swedish study has shown that the median age among women infected with ESBL-producing bacteria has decreased from 62 to 52 y in just five years, indicating a shifting epidemiological picture [10]. These risk factors are especially important to consider when treating patients with severe septicemia or septic shock empirically.

Even though the prevalence of ESBL-producing bacteria in Sweden is low in an international perspective, there has been a dramatic increase in the number of reported cases since 2007. In 2013 the number of reported cases were 8132, compared to the 7225 reported cases the previous year [11]. *European Centre for Disease Prevention and Control* (ECDC) registers and presents country prevalence data for resistant bacteria among invasive isolates, including *Escherichia coli* with resistance to third-generation cephalosporins from 30 European countries. The prevalence in Sweden was 4.4 % in 2012, compared to some parts of southern Europe with a prevalence as high as 38.1 % [12]. The prevalence of ESBL-carriage in the general population is not well-known, however. A Swedish study estimating the prevalence of ESBL-carriage among patients at Primary Health Care Units, which may represent a relatively healthy population, saw a rise from 2.1 to 3 % 2010, compared to various hospital wards where the prevalence increased from 1.8 % in 2008 to 6.8 % in 2010 [13]. Another Swedish study investigated the prevalence of ESBL-carriage among patients admitted to a surgery ward and found it to be 5 % [14]. Studies of faecal carriage of ESBL-producing *Enterobacteriaceae* in nursing homes in different countries have showed a wide range of prevalence estimates, ranging from 3 % (Sweden), 6.2 % (Belgium), and 11.2 % (Germany) to 40.5 % in Northern Ireland [8, 15–17].

Infections with ESBL-producing *Enterobacteriaceae* have dire clinical and economical consequences, including higher mortality, higher costs and prolonged hospital stays [18–21]. The major risk associated with colonization is progress to infection among hospitalized patients [22]. This cohort also serves as a reservoir for ESBL-producing *Enterobacteriaceae* [22–24], increasing the risk of spread to other patients, especially when there is poor adherence to hygiene routines [22, 23, 25, 26]. A Swedish study analyzed the ESBL-producing *Enterobacteriaceae* in a nursing home setting via pulsed-field gel electrophoresis (PFGE) and the result indicated a person-to-person transmission via the staff’s hands or clothes [15]. A further risk for patients infected with ESBL-producing *Enterobacteriaceae* is receiving inadequate empirical antibiotic treatment [27], which is the main reason behind the higher mortality in this group.

Only elderly that are requiring significantly care 24/7 are staying at nursing homes in Sweden since we have a well-developed tradition to take care of elderly at their own homes as long as possible. At nursing homes in Sweden there are usually nurses during daytime and doctors linked to the homes. The aim of this study was to investigate the prevalence of fecal ESBL-carriage in a nursing home setting and compare it with a corresponding population of elderly that still live in their own homes.

## Methods

### Study design

The study was designed as a cross-sectional comparison study in February to April 2014 to investigate the prevalence of fecal carriage of ESBL-producing *Enterobacteriaceae* in a nursing home setting compared with elderly living in their own homes. The study was carried out in the cities of Malmö and Lund in Sweden. In Malmö and Lund there are 59 and 16 nursing homes, respectively. Nursing homes that exclusively housed residents with dementia were excluded. A total of 30 nursing homes were contacted. In each of these nursing homes, a contact person (usually a nurse) provided a list of potential participants with the ability to provide informed consent. Elderly that had stayed at the nursing home of one month or more were eligible for inclusion. Each resident had a room of his or her own with separate toilet and shower.

Elderly with an age > 60 y living in their own homes were approached on a total of 8 meeting places for elderly. Ethical approval for the study was obtained from the Research Ethics Committee of the University of Lund 2013/898.

### Data collection

Rectal bacterial samples were acquired using a rectal swab pin (eSwab, Copan, Brescia, Italy). In the nursing homes, the samples were collected by a medical student. In the meeting places for the elderly the participants were collecting the samples themselves after a standardized instruction. One sample per person was obtained.

Every participant answered a standardized questionnaire regarding foreign travel, treatment with antibiotics, surgical procedures or hospital stay the last six months and prevalence of chronic heart disease, COPD, other chronic lung disease, neurological disease and stroke, diabetes mellitus, autoimmune disease, active cancer, hematological disease, liver failure, kidney failure, immunosuppressive treatment corresponding a daily dose of ≥ 5 mg prednisolone and length of stay at the nursing home. When needed, this information was obtained from the nurse in charge.

### Microbiological methods

All samples were transported in Amies medium (Copan, Brescia, Italy) to the Laboratory of Microbiology at Malmö University Hospital within 24 h. The samples were inoculated on agar plates with medium selective for cephalosporin resistance (ChromID ESBL, Bio-Mérieux™). All isolates obtained from these plates were further examined for ESBL production through synergy testing on Müller-Hinton agar plate (Oxoid Thermo Fischer) with discs containing cefpodoxime and ESBL- and AmpC-inhibitor (AmpC & ESBL Detection Set, Mast Group Ltd, Meyerside, UK). Antimicrobial susceptibility testing was performed on the ESBL-positive isolates using the disk diffusion method according to the Swedish Reference Group of Antibiotics method [28, 29]. The species determination was performed using MALDI-TOF. MIC determination was performed using E-test strips (Biomerieux, Marcy-l'Étoile, France).

### Statistics

Statistical analysis was performed using IBM SPSS version 22 (IBM Corporation, New York, USA). The analysis of risk factors was performed using Pearson’s $\boldsymbol{\chi}$^2^ or Fischer’s exact test, and 95 % confidence intervals were calculated using the method of Clopper and Pearson. Mann—Whitney *U* test was performed calculating p-values in age between both groups. An unadjusted *p*-value < 0.05 was considered statistically significant.

## Results

A total of 30 nursing homes were approached, 18 in Malmö and 12 in Lund. Of these, ten decided to participate, seven in Malmö and three in Lund. A total of 91 residents were recruited and subsequently screened. Ten residents denied participation after the information about the study was given. In the group of elderly living at home a total of 69 individuals were screened after they accepted participation. A total of 160 people were screened.

### Descriptive data

The median age in the nursing home cohort and control cohort was 89 and 78 years, respectively. The median age of the two groups together was 85 years. There was a female dominance in both groups, 69 and 74 % respectively. The descriptive characteristics and the results from the questionnaire in the two cohorts is shown in Table 1 In the nursing home cohort, the univariate analysis revealed significantly more hospital stays and recent antibiotic treatments, as well as higher proportions of chronic heart disease, COPD and neurological diseases. There was a difference in age where occupants at nursing homes were significantly older than study participants living in their own homes (*p* < 0.0001). No age differences were seen between ESBL and non-ESBL carriers for the whole study population, nor within the nursing-home or the non-nursing home group. However, international travel was significantly more frequent among the elderly living in their own homes (*p* = <0.001, Table 1).

**Table 1:** Demographics and risk factors

Variable	Nursing home (*n* = 91)	Living at home (*n* = 69)	*P*-value
Age (years)	65–102	60–98	–
Age median (years)	89	78	–
Female	63 (69 %)	51 (74 %)	–
Male	28 (31 %)	18 (26 %)	–
Hospital stay^a^	15 (16.5 %)	3 (4.3 %)	0,016
Surgery^a^	2 (2.2 %)	2 (2.9 %)	1
Foreign travel^a^	1 (1.1 %)	23 (33.3 %)	<0,001
Hospital stay abroad^a^	0	0	–
Chronic heart disease	31 (34.1 %)	11 (15.9 %)	0,01
COPD	10 (11.0 %)	1 (1.4 %)	0,024
Other chronic lung disease	5 (5.5 %)	4 (5.8 %)	1.00
Neurological disease, stroke	23 (25.3 %)	6 (8.7 %)	0.007
Diabetes mellitus	7 (7.7 %)	10 (14.5 %)	0.167
Autoimmune disease	8 (8.8 %)	3 (4.3 %)	0.353
Cancer	10 (11.0 %)	2 (2.9 %)	0.054
Haematological disease	1 (1.1 %)	0	1.00
Liver failure	1 (1.1 %)	0	1.00
Kidney failure	3 (3.3 %)	0	0.26
Immunosuppressive treatment^b^	0	0	–
Antibiotic treatment^a^	25 (27.5 %)	5 (7.2 %)	0.001

^a^The past six months ^b^Corresponding Prednisolon > 5 mg/day

### Prevalence of ESBL-carriage

The total prevalence of faecal carriage of ESBL-producing *Enterobacteriaceae* was 10 % (16/160; 95 % CI 5.8–15.7 %). In the nursing home cohort ten residents were ESBL-carriers, i.e. 11 % (95 % CI 5.9–19.2 %), and in the control cohort six individuals tested positive (8.7 % (95 % CI 3.7–18 The median age for the ESBL-carriers in the respective cohorts were 90 and 70.5 years. The majority of the carriers of ESBL-positive bacteria were women, 69 %, which is representative for the two groups (Table 2). The difference in prevalence between the two groups is not statistically significant (*p* = 0.632). In four of the ten nursing homes ESBL-producing bacteria were not isolated at all.

**Table 2:** Characteristics ESBL-positive (*n* = 16)

Participants	Nursing home	Living at home	Total
Female *n* (%)	7 (70)	4 (67)	11 (69)
Male *n* (%)	3 (30)	2 (33)	5 (31)
Total *n* (%)	10 (63)	6 (37)	16 (100)
Age (years)	75–97	67–81	67–97
Age (years) median	90	70,5	83,5

### Information on isolated bacteria

All of the isolates of the ESBL-producing *Enterobacteriaceae* were *E. coli.* One person was carrying two different isolates with different resistance pattern. All of the ESBL-producing isolates (*n* = 17) were susceptible to imipenem and amikacin. The resistance pattern of the strains is shown in Table 3. Nine out of the 17 isolates were resistant against ≥ 2 antibiotic classes in addition to betalactam antibiotics.

**Table 3:** Antibiotic resistance ESBL-producing *E. coli*-isolates (*n* = 17)

Antibiotic	Resistance in ESBL-producing *E.coli*-isolates (*n* = 17)
Gentamicin	24 %
Tobramycin	35 %
Amikacin	0 %
Imipenem	0 %
Trimethoprim-sulfametoxazole	77 %
Ciprofloxacin	59 %
Piperacillin-Tazobactam	6 %

### Risk factors for carriage of ESBL-producing E. Coli

Six out of 16 ESBL-carriers had travelled abroad in the past 6 months and five of these individuals were elderly living in their own homes. International travel was the only statistically significant association with ESBL-carriage (*p* = 0.017). Six persons had received antibiotic treatment in the past 6 months, all of them residents in nursing homes. For one of the ESBL-carriers, no apparent risk factor for ESBL-carriage was identified.

## Discussion

In this cross-sectional comparison study we found no difference in the prevalence of ESBL-producing *Enterobacteriaceae* among nursing home residents and the elderly living in their own homes (11 and 8.7 % respectively). We found that a total of 10 % were ESBL-carriers among all of the elderly in the study, which was higher than expected.

Our investigation has limitations. We did not perform PFGE, rep-PCR or MLST on isolates to examine the possibility of spread of the ESBL-producing *Enterobacteriaceae* between the elderly. However, an analysis of the respective resistance patterns was performed, and it showed widespread resistance to trimethoprim-sulfametoxazole and ciprofloxacin, two widely used antibiotics against common infections like UTIs. Another limitation is that only faecal sample per participant was collected, and an intermittent secretion of ESBL-producing bacteria could theoretically have been missed [17]. In total 160 persons were screened, a higher number would have increased the statistical power and perhaps the results could have been different. We don’t think so due to the fact that the healthy elderly is travelling a lot (in Sweden) and this seems to be of just as high importance to become rectal carrier of ESBL: s as staying at a nursing home. However, the risk for a type II error cannot be fully excluded in this material.

The two major reasons for not participate in the study was due to shortage of staff and a high ratio of residents suffering from dementia. The shortage of staff was an argument from some of the nursing homes that declined to participate, but we haven’t performed a survey over the staffing levels in the actual nursing homes. The size of the nursing home were not an important factor regarding participation in the study. Rooney et al. have hypothesized that nursing homes that participate in this kind of study are the ones that are sufficiently staffed and therefore are better equipped to observe hygiene routines [17]. In line with this, Andersson et al. reports that lack of time is the main reason why infection preventing routines are not followed [15], which increases the risk of spreading potential ESBL-producing bacteria via the staff’s hands or clothes [9, 15]. Only 91 of 434 residents in the nursing homes were screened. The main reasons for this loss was dementia diagnosis, but also severe illness and immobilization of the residents. Immobility, comorbidity and severe illness are risk factors for colonization with ESBL-producing *Enterobacteriaceae*, and not being able to include the residents with these risk factors might affect the result [4, 8]. In the group of elderly staying at home, when we finally found them there were only a few who declined participation so we did not perform any non-response analyzation.

Many nursing home residents per definition have risk factors for ESBL colonization and living at a nursing home is a risk factor per se [2, 4]. In this study, the ESBL-positive residents at nursing homes had between two and four established risk factors for colonization. Assessment of risk factors is therefore an important instrument to find patients who could be at risk of being colonized with ESBL-producing *Enterobacteriaceae*.

Nine of the 17 ESBL-positive isolates were resistant to ≥ 2 antibiotic classes in addition to betalactam antibiotics, which may complicate the treatment of common infections. The most common findings were resistance to ciprofloxacin and trimethoprim-sulfametoxazole, both antibiotics commonly used against UTIs and one has to consider the choice of antibiotics against this condition to get full effect and not to drive the resistance.

There was a female dominance in both study populations, and this corresponds partly with the representation of gender in Sweden among the elderly: in 2013, 60 % of individuals in the ages between 80 and 89 years [30] were female. The difference in age between the ESBL-positive in the two groups were 20 years (median age 90 and 70.5 years respectively), which in part reflects the difference in age between the two populations, but it also suggests the co-existence of different categories of individuals that become colonized with ESBL-producing *Enterobacteriaceae*, with different risk factors. Brolund et al. has shown that the median age of infected female patients in Sweden has decreased from 62 to 52 y between 2007 and 2011, which indicates a higher number of younger, healthier people being colonized with ESBL-producing *Enterobacteriaceae* through community-acquisition, which is a shift from earlier clinical picture where the nosocomial setting with elderly patients with comorbidity dominated [10]. Five of the six elderly living at home that were ESBL-positive had foreign travel as risk factor, whereof four had travelled outside the Nordic countries. Foreign travel was the only statistically significant connection to ESBL-carriage (*p* = 0.017), and has in Sweden been reported to be a major risk factor for ESBL acquisition, [24].

To our knowledge there is only one earlier study of the prevalence of faecal carriage of ESBL-producing *Enterobacteriaceae* in a nursing home setting in Sweden 2008, and this showed a prevalence of 3 % [15]. Other European studies have shown prevalence numbers ranging from 6.2 to 40.5 % [8, 17]. These numbers, along with our result, indicates that nursing homes may act as a reservoir for ESBL-producing bacteria. These results should be taken in consideration when treating these groups of patients if they develop severe sepsis or septic shock.

## Conclusions

The prevalence of ESBL-producing *Enterobacteriaceae* in the nursing homes studied has increased, but not more than among the healthy elderly population in general. This depends mainly on the fact that there are different risk factors in these two groups, and foreign travel—an established risk factor—has become far more common among healthy elderly people, leading to an increased risk of being colonized with ESBL-producing *Enterobacteriaceae* when travelling to countries and areas where the prevalence is higher. The risk of infection caused by ESBL-producing *Enterobacteriaceae* also increases the risk of getting inadequate initial empirical antibiotic treatment, which in turn increases the mortality in severe infections [18, 20, 21]. Our study showed that 1 of 10 elderly people carry ESBL-producing *E.* coli, which questions the current empiric antibiotic treatment of elderly with certain risk factors [27]. It would be desirable in future studies to include the residents with dementia or immobilization, to have a fuller picture, and to have a better match between the two groups regarding age and risk factors to be able to help these vulnerable individuals in the future.

## Abbreviations

COPD: chronic obstructive pulmonary disease
ECDC: European Centre for Disease Prevention and Control
ESBL: extended-spectrum beta-lactamase
PFGE: pulsed-field gel electrophoresis
UTI: urinary tract infection
